# In Vitro Antimicrobial Efficacy Assessment of Ethanolic, Aqueous, and Dual Solvent Extracts of Mushroom *Ganoderma lucidum*: Genomic and Morphological Analysis

**DOI:** 10.3390/antibiotics13121109

**Published:** 2024-11-21

**Authors:** Akamu Ewunkem, Lydia Merrills, Zahirah Williams, Brittany Justice, Uchenna Iloghalu, Vera Williams, Dinesh Singh

**Affiliations:** 1 Department of Biological Sciences, Winston Salem State University, Winston-Salem, NC 27110, USA; justicebl@wssu.edu (B.J.); iloghaluub@wssu.edu (U.I.); singhd@wssu.edu (D.S.); 2 Department of Nursing, Winston Salem State University, Winston-Salem, NC 27110, USA; lmerrills123@rams.wssu.edu (L.M.); zwilliams222@rams.wssu.edu (Z.W.); 3 UNC Health Care Hillsborough, Hillsborough, NC 27278, USA; virgowheels@yahoo.com

**Keywords:** antimicrobials, mushrooms, lingzhi, *Ganoderma lucidum*, infectious diseases, bacteria

## Abstract

**Background:** Infectious diseases caused by bacteria are life-threating and are among the major causes of death in the world. Antibiotics have offered humans a new approach to infection control. Antibiotics are reckoned as the “magic bullets” for the fight against bacterial infections, therefore increasing life expectancy and decreasing mortality and morbidity. However, the overuse of antibiotics has resulted in the persistent growth of resistant bacterial pathogens. New antimicrobial approaches against resistant pathogens are being examined. Mushrooms seem to be a promising, and possibly more efficient, alternative method to that of conventional antimicrobials. This work aimed to investigate the phytochemical constituents and antimicrobial potential of ethanolic, aqueous, and dual solvent extracts of mushroom *Ganoderma lucidum*. **Methods:** The antimicrobial studies were carried out by broth dilution against Gram-positive *Staphylococcus aureus* and Gram-negative *Escherichia coli*. The present research work was also carried out to examine genomic changes associated with ethanolic, aqueous, and dual solvent extracts of *G. lucidum* in *S. aureus* and *E. coli*. **Results**: Our data quantitatively showed that all the extracts of *G. lucidum* were found to exhibit various degrees of antimicrobial effects against *S. aureus* and *E. coli* where the ethanolic extract exhibited the most potent antimicrobial activity. SEM images showed untreated cells with normal cell characteristics while, after treatment with extracts of *G. lucidum*, cells appeared damaged with irregular cell surfaces and cell wall defacement. The results of HPLC analysis showed that ethanolic and aqueous extract of *G. lucidum* consisted of beta[1-3] glucans, ganoderic acid, and triterpenoids. Genomic analysis identified selective sweeps in several genes associated with growth, biosynthesis transport, and stress. **Conclusions:** This study concludes that the extracts of three solvents of *G. lucidum* have antimicrobial activity against infectious bacteria causing morphological changes and the acquisition of mutations in genes. Therefore, the extracts of *G. lucidum* may be candidates for preventing infectious diseases in the future. *Ganoderma lucidum* mushroom is therefore a reliable source of antimicrobial agent that can be used against infectious diseases.

## 1. Introduction

Microbial infections represent a significant cause of human morbidity and mortality [1]. Abating the burden of morbidity and mortality due to infection is a dire global public health concern. Antibacterial therapeutic options have offered humans a dramatic new approach to fight against life-threatening infections [2]. Pharmaceutical companies fight against infections by producing a wide variety of new antimicrobial medications to prevent and treat infectious diseases in humans. These medications exhibit antimicrobial activity by destroying the pathogens’ cell walls, interfering with the ability of the pathogens to multiply or make essential molecules for survival [3]. However, despite these advancements, the inadvisable use of antimicrobial medications has led to the fast emergence of antimicrobial resistance [4]. Most pathogens have an incredulous ability to mutate and acquire resistance [5].

Antimicrobial resistance is a severe threat to human health around the world and is directly responsible for millions of deaths annually [6]. This has resulted in searching for effective antimicrobial agents from alternative natural resources. A considerable amount of research has focused on natural products, like mushrooms, as sources of antimicrobial agents to prevent and treat infectious diseases [7]. Mushrooms, nature’s miniature pharmaceutical factories, have been used for thousands of years against clinical diseases due to the presence of diverse medicinal compounds, notably triterpenoids, beta-glucans, flavonoids, coumarin, mannitol, and alkaloids [8,9]. Studying the medicinal compounds of different species of mushroom, for example, *Ganoderma* sp. is relevant in this context, where antibiotics resistance is a global health problem and can provide health benefit by managing diseases. Additionally, molecular, and morphological changes associated with extracts of *Ganoderma* sp. are essential for identifying potential targets to disrupt bacterial survival pathways, ultimately helping to manage infectious diseases more effectively. However, reports on molecular and morphological changes associated with extracts of *Ganoderma* sp. have never been fully investigated.

Among all mushrooms, *Ganoderma lucidum* (also known as lingzhi) is one of the best-known and most studied curative mushrooms. In the Chinese language, lingzhi translates to “mushroom of immortality”, “divine mushroom”, or “magic fungus”. Additionally, lingzhi is perceived as the “herb of spiritual potency”, epitomizing success, well-being, divine power, and longevity [10]. In Asia, lingzhi is living up to its name, where it is traditionally prepared as hot water extracts. Hot beverages are imbibed as tea to aid heart health, support blood sugar reduction, enhance the immune system, reduce stress, improve sleep, and lessen fatigue [11,12,13,14]. Researchers are increasingly finding that *G. lucidum* or lingzhi possess other potential health benefits to ward off diseases and improve health.

*G. lucidum* is widely used to treat and prevent diseases owing to the presence of a plethora of active molecules that may act alone or in combinations to disrupt vital structural or cellular processes [15,16,17]. Several studies have investigated the antimicrobial potential of *G. lucidum* extracts [8,18,19,20,21,22]. However, each study used a given extraction method such as alcoholic and/or aqueous extracts. To the best of our knowledge, there are no studies comparing the antimicrobial potential of three extraction solvents obtained from *G. lucidum*. Preliminary studies utilizing HPLC in our lab revealed the presence of beta[1-3] glucans, ganoderic acids, triterpenoids, and other bioactive compounds known to exhibit antimicrobial activities [21]. *G. lucidum* is an important mushroom species to study for its valuable source of bioactivity and can be used to fight off antibiotic-resistant bacteria [18,19,20,21]. Therefore, the aim of this study was to determine the bioactive compounds of solvent extracts of *G. lucidum* using high-performance liquid chromatography (HPLC) and to examine their antimicrobial potential against *Staphylococcus aureus* and *Escherichia coli*. Furthermore, scanning electron microscopy (SEM) was employed to reveal morphological changes of bacterial cells that occurred upon treatment with the solvent extracts of *G. lucidum* and whole-genome sequencing analysis of the treated *S. aureus* and *E. coli* was performed to understand the mutations associated with the solvent extracts of *G. lucidum*, which may offer the potential to predict antimicrobial susceptibility. To the best of our knowledge, this is the first study to reveal ultrastructural changes and attempt to establish the possible targets of three extracts of *G. lucidum* on bacterial cells. *S. aureus* and *E. coli* were chosen for this study because their differential cell wall structures can divulge important information about how *G. lucidum* interacts with bacteria.

## 2. Materials and Method

### 2.1. Collection, Identification of Lingzhi

In August 2023, wild mushrooms, *Ganoderma lucidum* (Lingzhi), were collected from their natural habitats in Winston Salem, NC, USA. The demonstration samples were preserved in the Antimicrobial and Genomics Lab in the Department of Biological Sciences, Winston Salem State University, where standard keys were used to identify the mushrooms.

### 2.2. Preparation of Lingzhi Mushroom Extracts

**Figure 1:**
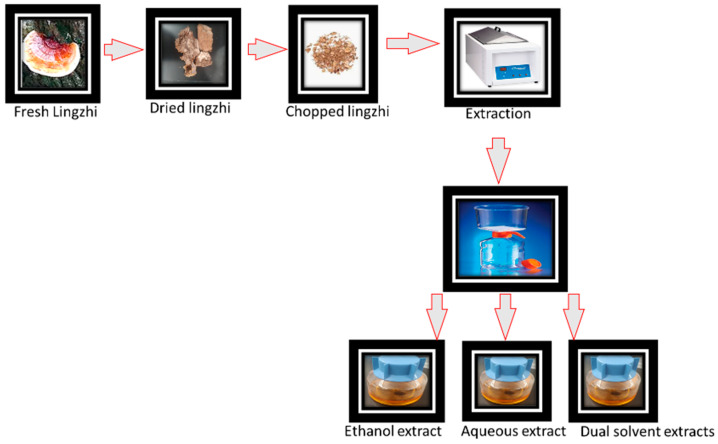
Extraction process of *G. lucidum* mushroom.

Fresh *G. lucidum* mushrooms were harvested and dried immediately. The harvested mushrooms were placed in a dehydrator (Fisher Scientific, Hampton, NH, USA) with no heat for three days. Subsequently, after drying, they were chopped and pulverized to particulates no larger than 2 mm and stored in double bags prior to beginning the extraction process. Mushroom extraction was carried out using 80% ethanol (Fisher Scientific, Hampton, NH, USA) and distill water as solvents. A 100 g quantity of pulverized mushroom was mixed with 0.5 L of each solvent in a conical flask (Fisher Scientific, NH, USA) at 50 °C and was shaken using an incubator shaker (Fisher Scientific, Hampton, New, NH, USA) at 150 rpm for 48 h. The extracts were centrifuged at 3000 rpm for 10 min and filtered to separate the organic matter through a Corning^®^ bottle-top vacuum filter system (Corning, NY, USA) at room temperature. Dual solvent (or double) extraction was prepared by initial extraction in ethanol at 50 °C for 48 h and finally in an equal volume of distilled water at 50 °C temperature for 48 h. The extract was then filtered as earlier described. All the extracts were stored at 4 °C in 1 L amber bottles (Fisher Scientific, Hampton, NH, USA) until used in the tests. The workflow of the extraction technique is shown in Figure 1.

### 2.3. High-Performance Liquid Chromatography (HPLC) Analysis

HPLC was carried out at Chemistry Laboratory in the Department of Chemistry, Winston Salem State University using Agilent 1100 series (Santa Clara, CA, USA) with a C18 column (4.6 mm × 250 mm i.d., 5 μm) at 35 °C for estimation of bioactive components in aqueous and ethanol extracts of *G. lucidum* mushroom. The mobile phase consisted of HPLC water (A) and acetonitrile (B) at a flow rate of 1 mL/min. The multi-wavelength detector was monitored at 280 nm.

### 2.4. Bacterial Strains and Growth Condition

The antibacterial potency of each mushroom extract was evaluated using Gram-negative *Escherichia coli* ATCC# 25922 and *Staphylococcus aureus* ATCC# 25923 procured from the Department of Biological Sciences, Winston Salem State University, Winston Salem, NC, USA. The tested bacteria strains were cultured overnight in nutrient broth (Fisher Scientific, NH, USA) at 37 °C and 160 rpm. The inoculum size of each bacterial strain was adjusted to a concentration of 1.5 × 10^8^ CFU/mL by comparing with 0.5 McFarland standards (Fisher Scientific, Hampton, NH, USA).

### 2.5. Antimicrobial Testing

Antimicrobial effects of ethanol extract, aqueous, and dual or double solvent extracts of *G. lucidum* against *E. coli* and *S. aureus* were investigated using the broth microdilution method in sterile 96-well plates (Fisher Scientific, Hampton, NH, USA), which were loaded with 100 µL of each extracted dilution (0–30%) into each well. Bacterial suspensions (10 µL) containing 10 × 10^3^ CFU were added to each well. Furthermore, positive control (without extract) and negative control (no inoculums) were also added to each well. The 96-well plates (Fisher Scientific, Hampton, NH, USA) were incubated at 37 °C, 160 rpm for 24 h. The growth of all the tested microorganisms was assessed by measuring turbidity at 600 nm for hours 0, 3, and 24 h, using a Glomax multi-plate reader (Promega, Madison, WI, USA). Examination of the morphological changes in *E. coli* and *S. aureus* treated with 30% of each extract after 24 h was performed using SEM (JEOL JSM-IT800 HL, JEOL Ltd, Akishima, Japan) following the methods described by Ewunkem et al. [22] and Ewunkem et al. [23].

### 2.6. Genomic Analysis

Isolation and sequencing of bacterial genomic DNA were performed at SeqCoast Genomics, Portsmouth, NH, USA. SeqCoast Genomics is an expert-guided genomics center offering microbial and small genome sequencing and bioinformatic analysis services. Briefly, after 24 h of exposure to the extracts, the suspension cells were transferred to sterile 1.5 mL centrifuge tubes (Fisher Scientific, Hampton, NH, USA). The centrifuge tubes were appropriately labeled and centrifuged at the highest speed for 1 min at 1600× *g*. The supernatant from each centrifuge tube was carefully removed without disturbing the pellet. The microcentrifuge tubes were immediately placed in a Styrofoam container and shipped overnight on dry ice to SeqCoast Genomics for whole-genome sequencing. DNA was extracted using the DNeasy 96 PowerSoil Pro QIAcube HT Kit (Qiagen 47021, Hilden, Germany) as per manufacturer instructions. Mechanical lysis was performed using MagMAX Microbiome bead beating tubes (Thermo Fisher Scientific, Waltham, MA, USA). Samples were prepared for whole genome sequencing as described by Ewunkem et al. [24]. The algorithm computes frequency by the number of reads that contain the de novo mutation of the bacterial cells exposed to *G. lucidum* extracts and the untreated cells (controls). A quality check for raw reads was performed with FASTQC, and the reads were trimmed and paired using Trimmomatic (version 0.39.0) and Breseq (version 0.37.0) [25].

### 2.7. Statistical Analysis

All the graphs, calculations, and statistical analyses were performed using GraphPad Prism software version 8.0 (GraphPad Software, San Diego, CA, USA). Statistical significance was defined by a *p* value of less than 0.05.

## 3. Results

### 3.1. HPLC of Aqueous and Ethanol Extracts of Lingzhi

**Figure 2:**
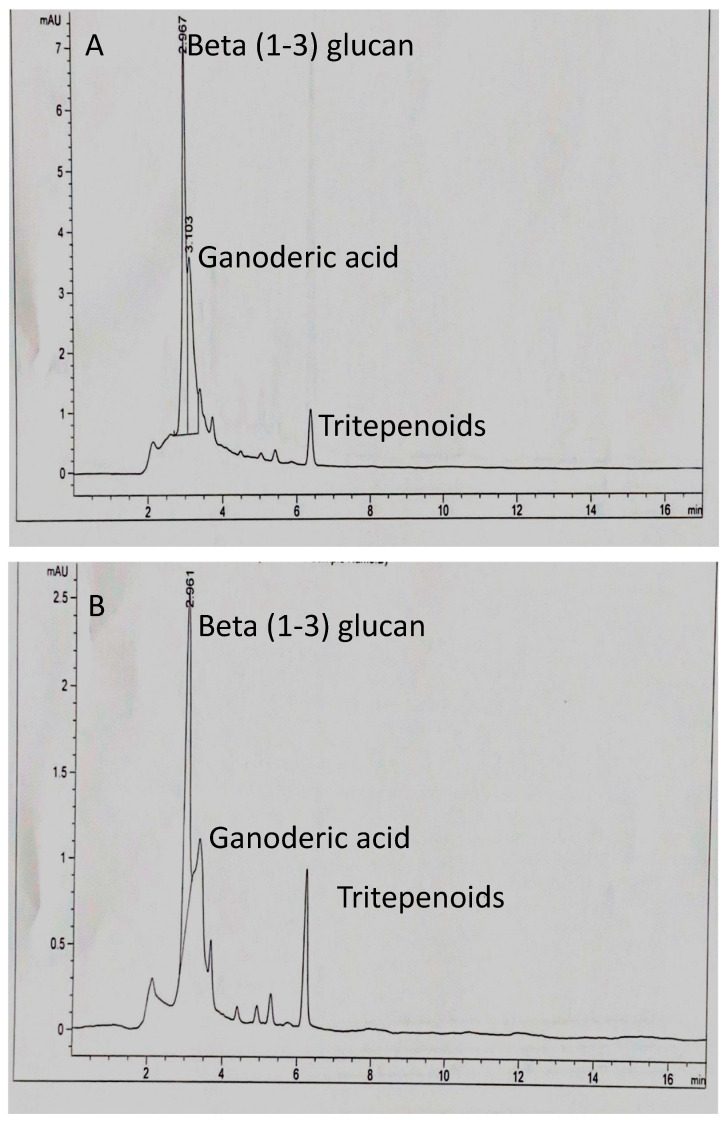
HPLC profile of ethanol (**A**) and aqueous (**B**) extracts derived from *G. lucidum* and the identified compounds/retention time.

HPLC analysis of aqueous and ethanol extracts of *G. lucidum* extracts was carried out to identify the bioactive compounds (Figure 2). The results showed qualitative profiles of both extracts of *G. lucidum*, which were composed of bioactive compounds. In addition, the chromatogram revealed that the aqueous and ethanol extracts showed a sharp peak at a retention time of 2.967 min (Figure 2A) and 1.961 min for ethanol and aqueous, respectively (Figure 2B). Three main bioactive compounds, (beta 1-3) glucans, ganoderic acid, and triterpenoids, were identified in the ethanol (Figure 2A) and aqueous extracts (Figure 2B). Based on the height of the peak, beta (1-3) glucan was the most abundant, followed by ganoderic acid.

### 3.2. Broth Microdilution Assay

**Figure 3:**
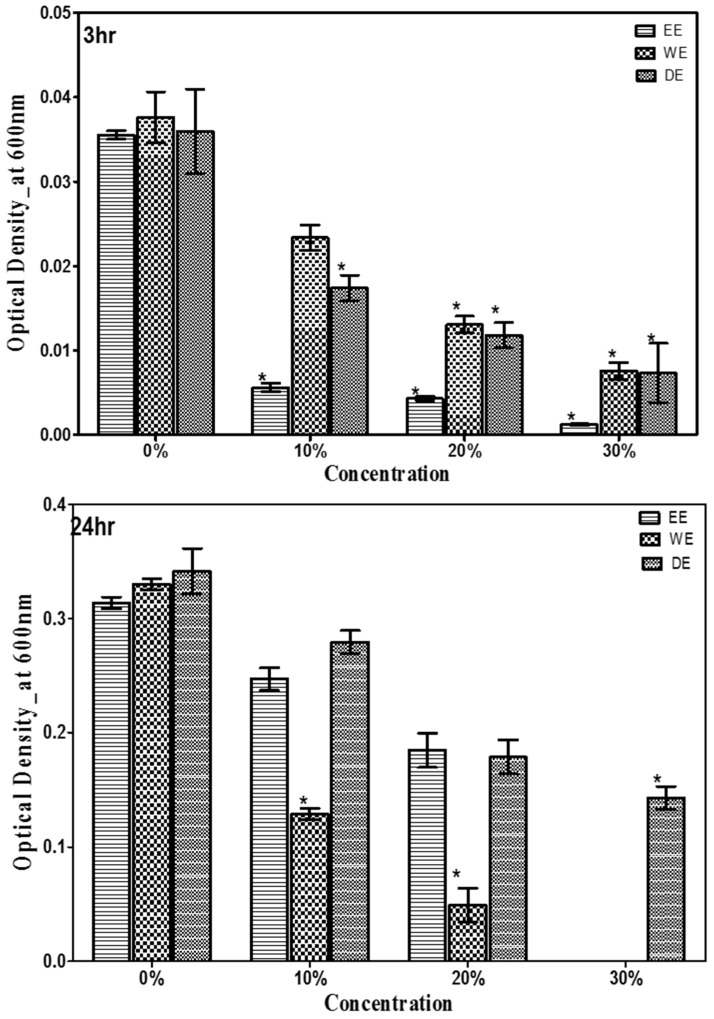
Antimicrobial activity of ethanolic, aqueous, and dual solvent extractions of *G. lucidum* against *E. coli* after 3 h and 24 h of incubation. The growth of *E. coli* was measured by a spectrophotometer at 600 nm wavelength. EE = ethanol extract; WE = water extract or aqueous extract; DE = dual extract. Asterisk (*) indicates significant difference compared with control (*p* < 0.05).

**Figure 4:**
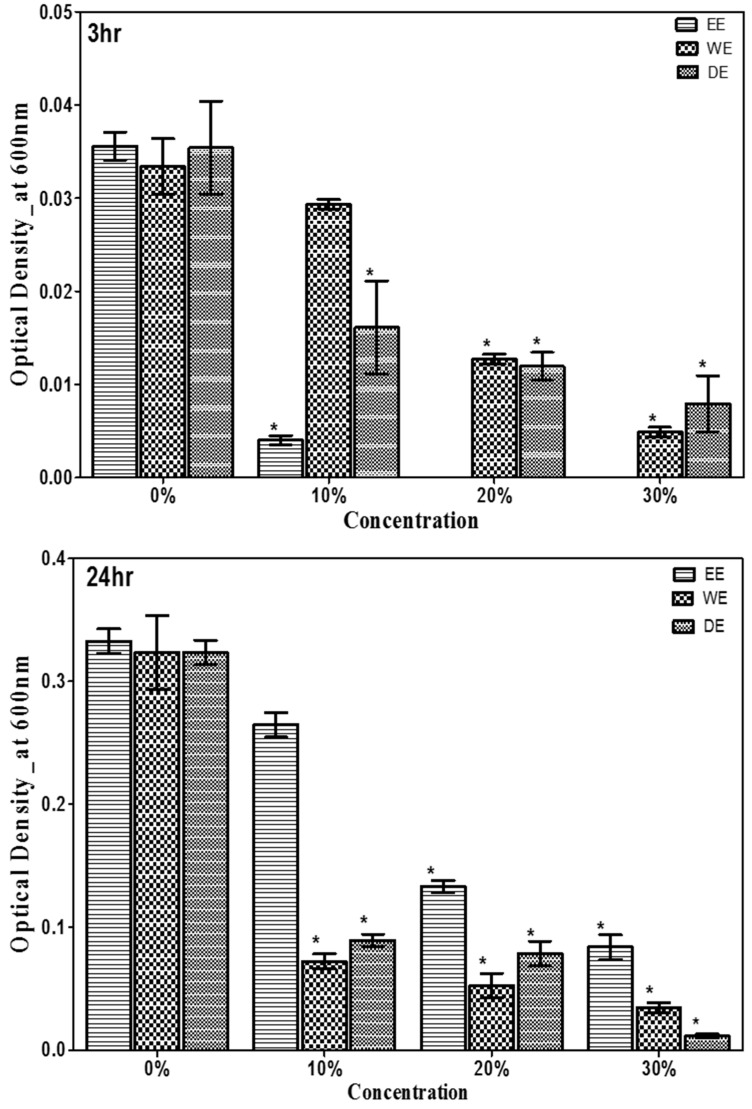
Antimicrobial activity of ethanol, water, and dual solvent extractions of *G. lucidum* against *S. aureus* after 3 h and 24 h of incubation. The growth of *S. aureus* was measured by a spectrophotometer at 600 nm wavelength. EE = ethanol extract; WE = water extract or aqueous extract; DE = dual extract. Asterisk (*) indicates significant difference compared with control (*p* < 0.05).

The antimicrobial activity of *G. lucidum* mushroom was investigated against *Escherichia coli* and *Staphylococcus aureus* by using three solvent extracts (ethanol, aqueous, and dual) after 3 and 24 h of incubation are shown in Figure 3 and Figure 4. All the three solvent extracts of *G. lucidum* were found to have antibacterial effects. The effectiveness of these extracts was observed within the first 3 h of incubation and continued to increase gradually at landmark until 24 h. In general, each solvent extract inhibited *E. coli* and *S. aureus* in a dose-responsive manner (Figure 3 and Figure 4). Each of the tested concentrations of *G. lucidum* exhibited a significant (*p* < 0.05) effect over the control. At each concentration, the optical density of *E. coli* and *S. aureus* decreased significantly (*p* < 0.05) by at least 25%. Ethanol extract significantly (*p* < 0.05) showed the most potent antimicrobial activity against *E. coli* and *S. aureus* (Figure 3 and Figure 4). The dual extract of *G. lucidum* had approximately equal antimicrobial activity against *E. coli* and *S. aureus* (Figure 3 and Figure 4). Collectively, ethanolic, aqueous, and dual solvent extractions of *G. lucidum* mushroom revealed varied antimicrobial activity against *E. coli* and *S. aureus.* The highest tested concentration acted lethal to *E. coli* and *S. aureus.*

### 3.3. Scanning Electron Microscopy (SEM) Observation

**Figure 5:**
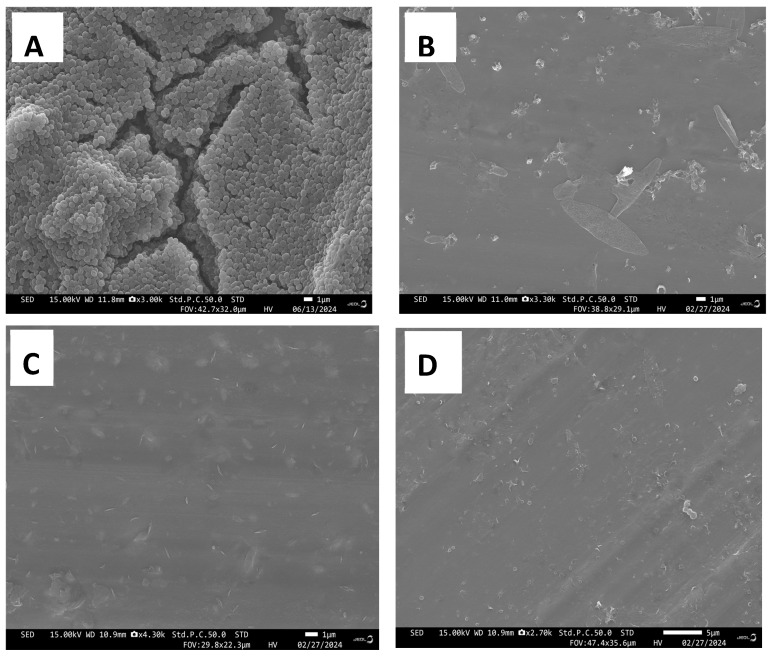
Scanning electron microscopy micrographs of *S. aureus* and control groups after incubation with various *G. lucidum* extracts at 24 h. (**A**) *S. aureus* control cell with normal surface; (**B**) ethanol extract-treated *S. aureus* with surface irregularities; (**C**) water extract-treated *S. aureus* treated with surface irregularities; (**D**) dual solvent extraction-treated *S. aureus* with surface irregularities.

**Figure 6:**
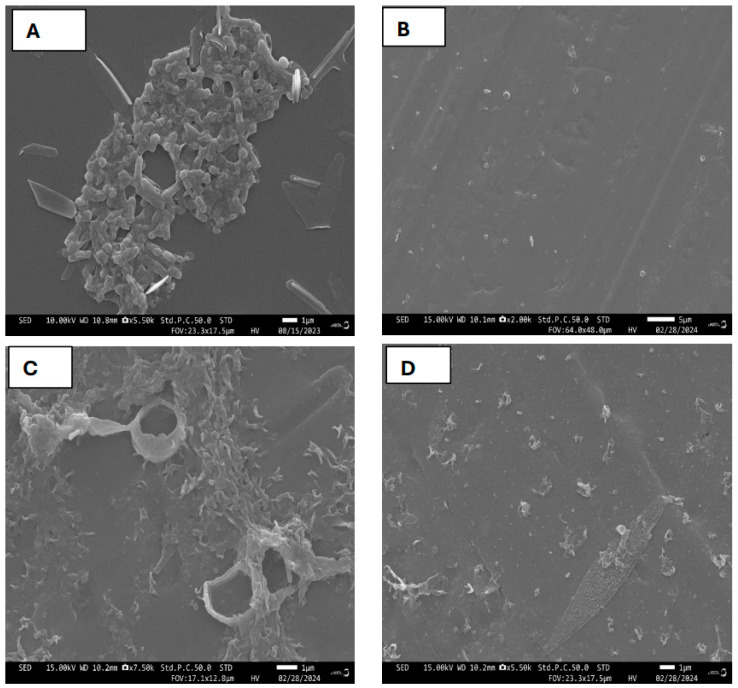
Scanning electron microscopy micrographs of *E. coli* and control groups after incubation with various *G. lucidum* extracts at 24 h. (**A**) *E. coli* control cell with normal surface; (**B**) ethanol extract-treated *E. coli* with surface irregularities; (**C**) water extract-treated *E. coli* with surface irregularities; (**D**) dual solvent extraction-treated *E. coli* with surface irregularities.

Scanning electron microscopy (SEM) analysis provided additional evidence that *G. lucidum* extracts effectively inhibited microbial growth, which was associated with cellular structural impairment with control cells exhibiting normal shape (Figure 5 and Figure 6). Under normal conditions, *S. aureus* showed normal cell characteristics with cocci shape and intake peptidoglycan layer. Following treatment with *G. lucidum* extracts, the cells appeared to be damaged with some irregularities (Figure 5). The membrane became rough, shrunken, and ruptured. Interestingly, all spherical-shape cells treated with aqueous extracts were changed to rod-shaped bacterial cells (Figure 5C). Untreated *E. coli* showed normal cell characteristics with a rod shape, while treatment with the *G. lucidum* extracts provoked cell surface irregularities (Figure 6). *E. coli* cells were wrinkled, shrunken, rough, and curved. Collectively, SEM exhibited that the *S. aureus* and *E. coli* experienced severe damage after being exposed to ethanol, aqueous, and dual extracts of *G. lucidum*.

### 3.4. Genomics Changes

The genomic variants found in the *S. aureus* control and treated cells are listed in Table 1, Table 2, Table 3 and Table 4. The following hard sweeps (new advantageous mutations) were observed in both the control and treated cells at 24 h incubation (Ewunkem et al. [24]): DUF1433 domain-containing protein (*KQ76_RS09235*); DNA-binding heme response regulator (*hssR*); alpha/beta hydrolase (*KQ76_RS13020*); ATP-binding protein (*KQ76_RS04770*); glutathione peroxidase (*KQ76_RS13475*); ribosome biogenesis GTPase (*ylqF*); ECF-type riboflavin transporter substrate-binding protein (*KQ76_RS13825*); tRNA uridine 5 carboxymethylaminomethyl(34) synthesis enzyme (*mnmG*); D lactate dehydrogenase (KQ76_RS12955); M23 family metallopeptidase/HAD IIB family hydrolase (*KQ76_RS11280/KQ76_RS11285*); BglG family transcription antiterminator (*KQ76_RS10985*); DNA binding heme response regulator (*hssR*); serine tRNA ligase/AzlC family ABC transporter permease (*serS/KQ76_RS00050*) hypothetical protein (*KQ76_RS09255*) (Table 1, Table 2, Table 3 and Table 4). Only the control cells displayed mutations in exonuclease SbcCD subunit D (*KQ76_RS09465*) and conserved phage C terminal domain-containing protein (*KQ76_RS07500*). The treated cells displayed a hard selective sweep in gluconokinase (*gntK*) ribosome biogenesis GTPase (*ylqF*), glutamate synthase large subunit (*gltB*), lipoyl synthase (*lipA*), and multicopper oxidase (*mco*), as observed in cells treated with ethanol (Table 2). Cells treated with aqueous extracts of *G. lucidum* displayed hard sweeps in glutamate synthase large subunit (*gltB*) (Table 3). Finally, cells treated with dual extracts of *G. lucidum* showed significant sweeps in Mn (2+)-dependent dipeptidase (*sapep),* BCCT family transporter (*KQ76_RS11175*), gluconokinase (*gntK*), nand efflux RND transporter permease subunit/lipid II:glycine glycyltransferase (*KQ76_RS11540/femX*). Furthermore, cells treated with ethanol and dual-extracted displayed mutations in ECF-type riboflavin transporter substrate-binding protein (*KQ76_RS13825*) (Table 2 and Table 4).

*E. coli* control and treated cells shared seven hard sweeps (Table 5, Table 6 and Table 7): intermembrane transport protein (*pqiB*), glucans biosynthesis protein (*mdoG*), invasion regulator (*sirB2*), histidinol dehydrogenase (*hisD*), and YtfJ family protein (*D1792_RS11465*). Only the control cells showed mutation in L lysine exporter LysO/aquaporin (*lysO/aqpZ*), PTS fructose transporter subunit IIC (*fryC*), DUF4756 family protein (*D1792_RS05680*), L lysine exporter LysO/aquaporin (*lysO/aqpZ*), phosphoglycerate dehydrogenase/SIS domain-containing protein (*D1792_RS10070/D1792_RS10075*), bifunctional chitinase/lysozyme (*chiA*), LysR family transcriptional regulator(*rcdB*), DnaA initiator associating protein (*diaA*), L lysine exporter LysO/aquaporin (*lysO/aqpZ*), LysR family transcriptional regulator (*rcdB*), and phosphoglycerate dehydrogenase/SIS domain-containing protein (*D1792_RS10070/D1792_RS10075*) (Table 5). *E. coli* cells treated with ethanol extracts of lingzhi showed hard selective sweeps in GntR family transcriptional regulator (*lgoR*) and serine protease autotransporter toxin Pic/hypothetical protein (*pic/D1792_RS20755*) (Table 6). The following significant polymorphisms were found in cells treated with dual extract of *G. lucidum*: helix-turn-helix transcriptional regulator (*D1792_RS02575*), two-component system sensor histidine kinase (*baeS*), and serine protease autotransporter toxin Pic/hypothetical protein (*pic/D1792_RS20755*) (Table 7). Unfortunately, the DNA sequencing reaction failed for *E. coli* treated with aqueous extract due to insufficient DNA. Most of the cells were dead after 24 h of incubation (Figure 2).

## 4. Discussion

Despite the vast diversity in antibacterial compounds, bacterial resistance to first-choice antibiotics is drastically increasing, resulting in the rise of life-threatening infections. Natural resources have been exploited in the last 100 years, and among them, mushrooms could be an alternative source of new antimicrobials [26]. *Ganoderma lucidum* (lingzhi) is a popular medicinal mushroom that has been used in Asian medicine because of its promoting effects on health and life expectancy [18,20,27,28,29,30,31]. However, the antimicrobial properties of three solvent extracts (aqueous, ethanol, and dual solvent extractions) of *G. lucidum* have never been evaluated for their antibacterial property against multidrug-resistant bacteria. Also, the genomic changes associated with aqueous, ethanol, and dual extracts of *G. lucidum* in multidrug-resistant bacteria have never been evaluated.

Given the above findings, there is a lack of information on genomic analysis and antimicrobial activity of aqueous, ethanol, and dual solvent extractions of *G. lucidum* against multidrug-resistant bacteria. Herein, we investigated the antimicrobial activity of *G. lucidum* extracts using three solvents, ethyl ethanol, aqueous, and dual (combined ethyl ethanol and aqueous), against *Staphylococcus aureus* and *Escherichia coli* at 3 h and 24 h of incubation. HPLC was carried out to identify bioactive compounds ethyl ethanol, aqueous, and dual solvent extracts of *G. lucidum*. Furthermore, scanning electron microscopy (SEM) was used to examine the ultrastructural changes in bacteria and genomic changes induced by ethyl ethanol, aqueous, and dual solvent extracts of *G. lucidum.*

All the *G. lucidum* extracts (ethyl ethanol, aqueous, and dual solvent extract) used in this study exhibited various degrees of antimicrobial activity against *S. aureus* and *E. coli* within the first 3 h of treatment and continued until 24 h in a concentration-dependent manner. The differences in antimicrobial activities of the *G. lucidum* extracts can be attributed to variations in their chemical constituents and the volatile nature of their components. Additionally, extracts from organic solvents are relatively more efficient in extracting antimicrobial compounds [32]. The findings of the current research revealed that ethanolic extract of *G. lucidum* showed a higher inhibitory effect on *E. coli* and *S. aureus* than aqueous and dual extracts. Our results are consistent with previous studies. Silva et al. [33] showed that ethanol extracts from several plant species showed superior activity against *E. coli*, *B*. *cereus*, *C. albicans*, and *C. parapsilosis* in water and hexane extract. Thouri et al. [34] demonstrated that ethanolic extract from Acacia and Eucalyptus was the most effective against *E. coli*. The antimicrobial activity of extracts of *G. lucidum* might be due to the presence of several bioactive compounds such as beta[1-3] glucans, ganoderic acid, and triterpenoids (Figure 2). The HPLC chromatogram proved that in both aqueous and ethanol extracts of *G. lucidum*, a major peak in β-glucan appeared at ~2.9 min, as shown in Figure 2. These results come as no surprise, since beta-1,3-Glucan is a major constituent of fungal cell walls [8]. Furthermore, beta-1,3-Glucan has been shown to have strong antioxidant, anti-inflammatory, and antimicrobial activities [8,20]. The solvent polarity of the solvent is known to significantly impact the quantity of the dissolved bioactive compound [35]. Ethanol extracts generally have more potent bioactive compounds due to their high polarity. Higher polarity can concentrate on a greater number of therapeutic products [36]. *G. lucidum* is a well-known medicinal mushroom and a potential source of many therapeutic substances such as lectins, polysaccharides (β-glucans), polysaccharide peptides, polysaccharide–protein complexes, lanostanoids, terpenoids, alkaloids, sterols, and phenolic-structured compounds [22,37,38]. The antimicrobial activity of ethanolic extracts can also be attributed to the inherent nature of antimicrobial properties such as denaturation and protein coagulation [39].

Gram-negative bacteria are typically less sensitive to mushroom extracts because of a membrane surrounding the peptidoglycan that limits the diffusion through its lipopolysaccharide (LPS) covering. LPS provides structural integrity, and a permeability barrier protects the bacterial cell from the entry of harmful or toxic substances [40]. Gram-positive bacteria lack this vital layer, which makes Gram-positive bacteria more sensitive to mushroom extracts than Gram-negative bacteria [41,42]. In this study, *E. coli* was more susceptible to the inhibitory action of all the extracts of *G. lucidum* than *S. aureus*. The explanation for the unexpected antimicrobial activity of *G. lucidum* against *E coli* and *S. aureus* is argued to be due to differences in structural modification between Gram-negative and Gram-positive bacteria. *S. aureus* and *E. coli* are Gram-positive and Gram-negative bacteria, respectively. Gram-positive bacteria have a large amount of peptidoglycan and no outer lipid membrane present while Gram-negative have a substantially thin peptidoglycan layer and an outer membrane lipid [43]. The thickening of the peptidoglycan prevents the extracts of *G. lucidum* from reaching and binding targets. This finding is supported by results of Juan et al. [44], who demonstrated that enlargement of the peptidoglycan allows for *S. aureus* to resist vancomycin. Furthermore, the thicker of the cell wall, the faster the growth recovery/suppressive effect of antimicrobials.

The antimicrobial mechanisms of *G. lucidum* are not entirely revealed and require further research. Nevertheless, several potential mechanisms have been proposed, such as the activation of reactive oxygen species (ROS) that damage bacterial DNA, proteins, and other cellular components causing oxidative stress and cellular death [45,46]. In addition, *G. lucidum* is known to inhibit bacterial growth by inhibiting replication, ATP, DNA synthesis, and changing metabolic processes [9]. This study examined the mechanism of action of the various solvent extracts of *G. lucidum* to assess its potential for use in evidence-based antimicrobial therapy. Scanning electron microscopy (SEM) was used to determine the morphological alteration after *S. aureus* and *E coli* cells were treated with ethanol, aqueous, and dual extracts from *G. lucidum*. Cells treated with extracts of *G. lucidum* showed some significant morphological changes. The cells appeared ruptured, with degradation of the cell walls, as was the case in previous studies where extracts of *Ocimum basilicum* and *Lentinus tigrinus* induced changes in the morphology of *Bacillus cereus*, *Pseudomonas aeruginosa*, *Shigella* sp., *Listeria monocytogenes*, *Staphylococcus aureus*, and *Escherichia coli* [47,48]. The degradation of the cell wall might be due to the diverse chemical composition of the extract and different target sites [22,49]. Our HPLC profile revealed similar peaks in aqueous and ethanol extracts that were identified to represent ganoderic acid, beta[1-3] glucans, and triterpenoids. These bioactive compounds might have damaged bacterial cells, disrupting vital cellular processes and essentially inhibiting bacterial growth.

However, further investigation into the mechanism of action of isolated pure compounds from each solvent extract of *G. lucidum* is required. Exposure of the tested bacteria to the three solvent extracts of *G. lucidum* might have induced mutations in genes that significantly affected their phenotype by altering the function protein encoded by these genes, causing the observable trait in the bacteria. Herein, we utilized whole-genome sequencing to identify mutations in *E. coli* and *S. aureus* that might impact their phenotype and antimicrobials.

Chromosomal mutations corresponding to antimicrobials of clinical importance were identified from whole-genome sequencing data. Here, we found mutations in *S. aureus* cells treated with extracts of *G. lucidum*. For example, genomic analysis of *S. aureus* cells treated showed mutations in genes involved in metabolism and synthesis that help bacteria evade the toxic effects of antimicrobials. These are linked to the following selective sweeps in mutations in gluconokinase (*gntK*), ribosome biogenesis GTPase (*ylqF*), glutamate synthase large subunit (*gltB*), lipoyl synthase (*lipA*), and multicopper oxidase (*mco*), as observed in cells treated with ethanol (Table 2): Glucokinase helps stabilize newly synthesized proteins [50]. Ribosome biogenesis GTPase links aspects of the cell cycle and metabolism with translation and coordinates connections between these aspects [51]. Glutamate synthase large subunit plays a key role in the ammonia assimilation pathways found in bacteria [52]. Lipoyl synthase (*LipA*) catalyzes the final step in the biosynthesis of the lipoyl cofactor [53]. Multicopper oxidases play a crucial role in copper detoxification in many bacteria [54].

Genomic analysis in *S. aureus* control and treated cells illustrated 14 selected weeps in genes associated with transport gene expression, peptide bond synthesis, ATP hydrolysis, protection, cellular function, and glycolysis, such as in the case of the following genes: [24] DUF1433 domain-containing protein (*KQ76_RS09235*); DNA-binding heme response regulator *(hssR*); alpha/beta hydrolase (*KQ76_RS13020*); ATP-binding protein (*KQ76_RS04770*); glutathione peroxidase (*KQ76_RS13475*); ribosome biogenesis GTPase *(ylqF*); ECF-type riboflavin transporter substrate-binding protein (*KQ76_RS13825*); tRNA uridine 5 carboxymethylaminomethyl (34) synthesis enzyme (*mnmG*); D lactate dehydrogenase (*KQ76_RS12955*); M23 family metallopeptidase/HAD IIB family hydrolase (*KQ76_RS11280/KQ76_RS11285*); BglG family transcription antiterminator (*KQ76_RS10985*); serine tRNA ligase/AzlC family ABC transporter permease (serS/KQ76_RS00050); hypothetical protein (*KQ76_RS09255*). Each of these genes plays a unique role in bacterial adaptation and cellular functions. DUF1433 domain-containing protein (*KQ76_RS09235*) has unknown function; DNA-binding heme response regulator *(hssR*) regulates DNA-binding activity and controls gene expression [55]; alpha/beta hydrolase (*KQ76_RS13020*) is responsible for the hydrolysis of ester and peptide bonds [56]; ATP-binding protein (*KQ76_RS04770*) is membrane-bound proteins that use the energy from ATP hydrolysis to move substrates across the cell membrane and play roles in nutrient uptake and in secretion of toxins and antimicrobial agents [57]; glutathione peroxidase (*KQ76_RS13475*) protects the cell from oxidative damage by reducing lipid hydroperoxides to alcohols and hydrogen peroxide to water [58]; ribosome biogenesis GTPase *(ylqF*) is involved in ribosome assembly, translation, and signal transduction [51]; ECF-type riboflavin transporter substrate-binding protein (*KQ76_RS13825*) is a transmembrane protein that help uptake micronutrients, such as B-type vitamins and cations, into cells [59]; tRNA uridine 5 carboxymethylaminomethyl (34) synthesis enzyme (*mnmG*) is involved in tRNA modification [60]; D lactate dehydrogenase (*KQ76_RS12955*) is necessary for glycolysis [61]; M23 family metallopeptidase/HAD IIB family hydrolase (*KQ76_RS11280/KQ76_RS11285*) is used by bacteria to lyse cell walls of other bacteria and nematodes [62]; BglG family transcription antiterminator (*KQ76_RS10985*) controls the expression of carbohydrate transporters [63]; serine tRNA ligase/AzlC family ABC transporter permease (serS/KQ76_RS00050) catalyzes the attachment of serine to tRNA(*Ser*) [64]; hypothetical protein (*KQ76_RS09255*) is associated peptidoglycan metabolism, cell wall organization, ATP hydrolysis, and outer membrane fluidity [65]. Two of these genes are associated with antibiotic resistance, such as in the case of the ATP-binding protein (*KQ76_RS04770*) and glutathione peroxidase (*KQ76_RS13475*), which play roles in nutrient uptake secretion of toxins, and antimicrobial agents protect the cell from oxidative damage [57,58], suggesting that antibiotics can be synthesized to target these genes in an attempt to treat staphylococcal infections. The control and treated cells share the same mutations because genes in both cells are inherently susceptible to mutations based on their location and function. These genes might have been found in the same loci or location of the genome and are involved in cellular functions such as growth, adaptation, transport, and secretions. The mutations in the control and treated cells were similar because the extracts of *G. lucidum* did not significantly induce mutations due to either low concentration or duration of the extracts. In addition, the extracts might have directly increased the chances of mutations in these vulnerable locations.

It is also important to note that two selective sweeps were found exclusively in the control cells: mutations in exonuclease SbcCD subunit D (*KQ76_RS09465*) and conserved phage C terminal domain-containing protein (*KQ76_RS07500*). Mutations in these genes play a role in DNA repair, environmental sensing, and regulating genes [66,67]. It can be argued that these two selective sweeps displayed in specific genes are associated with DNA repair, environmental sensing, and bacterial growth enhancement. Mutation typically occurs in untreated cells in the media because of intrinsic errors during DNA replication, resulting in changes in genetic sequence, and some mutations may randomly occur to become fixed mutations. The composition of the media (nutrient broth) used in our experiments typically consists of peptone, beef extract, sodium chloride, amino acids, vitamins, salts, and other nutrients. This composition could have contributed to *S. aureus* mutations by creating conditions that enhance promote growth and oxidative stress, thus increasing the natural mutation rate within a bacterial population.

Genes present in Gram-negative *E. coli* differ significantly from genes in Gram-positive *S. aureus* due to the significant structural differences in their cell walls. The additional outer membrane in Gram-negative bacteria could have led to variation in mutations in genes associated with cell wall synthesis, antibiotic resistance, and interaction with other cells. The structural distinction is also based on the difference in thickness peptidoglycan layer, where Gram-positive bacteria have a thick layer while Gram-negative bacteria have a thin layer. Both control and treated *E. coli* cells showed mutations in genes associated with growth, transport invasion and colonization, synthesis survival, and stress. For example, glucans biosynthesis protein (*mdoG*) is involved in microbial recognition [68]. The intermembrane transport protein (*pqiB*) is a membrane-embedded protein transport system that regulates transport proteins [69]. Histidinol dehydrogenase (hisD) catalyzes the terminal step in the biosynthesis of histidine essential for the survival of bacteria associated with several infections [70]. Invasion regulator (*sirB2*) is involved in invasion and colonization [71]. Histidine’s features make it very interesting for the study of new strategies aimed at developing novel classes of antibacterial agents. YtfJ family protein (*D1792_RS11465*), regulated by *RpoE*, an alternative sigma factor that regulates genes in response to extracellular stress [72]. As earlier mentioned, the control cells shared the same mutation as treated cells because the extracts of *G. lucidum* might not have significantly induced mutations.

The remaining mutations in the control cells showed evidence of protection against high concentrations of metals, transport of sugar, stress, and antibiotics. Specifically, these mutations occurred in lysine exporter LysO/aquaporin (*lysO/aqpZ*), which protects against high concentrations of heavy metals in the cytoplasm by exporting amino acids, lipids, and heavy metal ions [73]; PTS fructose transporter subunit IIC (*fryC*) is responsible for transporting sugar molecules across the inner bacterial membrane [74]; DUF4756 family protein (*D1792_RS05680*) is involved in bacterial stress responses and infections [75]; phosphoglycerate dehydrogenase/SIS domain-containing protein (D1792_RS10070/D1792_RS10075) catalyzes the first step in the biosynthesis of l-serine, a nonessential amino acid [76]; bifunctional chitinase/lysozyme (*chiA*) promotes colonization of the intestines [77]. LysR family transcriptional regulator (*rcdB*) affects antibiotic tolerance and virulence [78]; DnaA initiator-associating protein (*diaA*) plays a role in the initiation of chromosomal replication [79] and phosphoglycerate dehydrogenase/SIS domain-containing protein (D1792_RS10070/D1792_RS10075) plays a key role in the serine biosynthesis pathway (SSP) in bacteria [80]. The compositions of the culture media and long-term batch culture likely affected cellular function and subsequently induced mutations explained in the previous paragraphs. Treating these bacteria with antimicrobials can increase the rate at which they mutate (antibiotic-induced mutagenesis). This can help *E. coli* develop a defense mechanism against *G. lucidum* extracts, as described in the next paragraph.

Three different mutations were detected in *E. coli* cells treated with ethanol extract of *G. lucidum*. These mutations showed evidence in stress responses and production of metabolites, as in the case of transcriptional regulator (*lgoR*) [81] and serine protease autotransporter toxin Pic/hypothetical protein (*pic/D1792_RS20755*), which plays a role in the virulence and survival of pathogens [82]. Additionally, three significant mutations were found in cells treated with dual extract of *G. lucidum*: helix-turn-helix transcriptional regulator (*D1792_RS02575*) regulates gene expression, DNA repair, RNA metabolism, and protein–protein interaction [83]. Two-component system sensor histidine kinase (*baeS*) helps organisms’ sense and respond to environmental changes. Serine protease autotransporter toxin Pic/hypothetical protein (*pic/D1792_RS20755*) mediates acclimation to various environmental changes by coupling environmental cues to gene expression [84]. Mutations in these genes enable *E. coli* to adapt to challenging environments, making it difficult to treat infections caused by *E. coli.* This may require extended hospital stays and additional doctor visits.

Control and treated *E. coli* cells showed mutations in intermembrane transport protein (*pqiB*), glucans biosynthesis protein (*mdoG*), invasion regulator (*sirB2*), histidinol dehydrogenase (*hisD*), and YtfJ family protein (*D1792_RS11465*). Glucans biosynthesis protein (*mdoG*) is involved in microbial recognition [68]. Intermembrane transport protein (*pqiB*) is a membrane-embedded protein transport system that regulates transport proteins [69]. Invasion regulator (*sirB2*) is involved in invasion and colonization [71]. Histidinol dehydrogenase (*hisD*) catalyzes the terminal step in the biosynthesis of histidine essential for the survival of bacteria associated with several infections, M Monti et al. [70]. This feature makes it very interesting for the study of new strategies aimed at developing novel classes of antibacterial agents. YtfJ family protein (*D1792_RS11465*) is regulated by *RpoE*, an alternative sigma factor that regulates genes in response to extracellular stress [72].

## 5. Conclusions

Infectious diseases caused by pathogenic bacteria are responsible for high morbidity and mortality throughout the world. Antimicrobial agents, including antibiotics, have been used for millennia to manage or treat infectious diseases. However, pathogenic bacteria have acquired resistance in response to the development of these antimicrobial agents. Hence, there is a great desire for patients to use natural products with antimicrobial properties. This situation has forced scientists to research new antimicrobial substances that are effective against resistant strains to conventional treatments. Medicinal mushrooms are promising alternatives that provide safe and reliable benefits in treating infectious diseases. This study explored the antimicrobial activity of ethanol, aqueous, and dual solvent extractions (combined ethanol and aqueous extracts) from mushrooms *Ganoderma lucidum* against *S. aureus* and *E. coli* (examples of the two most frequent causes of many infectious diseases). Ethanol, aqueous, and dual solvent extractions of *G. lucidum* showed antimicrobial activity against *S. aureus* and *E. coli* due to several bioactive compounds. Ethanolic extract had the highest performance. Observed treated cells confirmed obvious changes in morphology in response to ethanol, aqueous, and dual solvent extractions from *G. lucidum*. Genomic results indicated that these cells showed mutations in genes essential for cellular functions and adaptation. This study may suggest that extracts from *G. lucidum* mushrooms are a potential source of many therapeutic and pharmaceutical products with significant health importance. The antimicrobial activity of aqueous, ethanol, and dual extract solvents of *G. lucidum* against common pathogenic bacteria such as *E. coli* and *S. aureus* makes *G. lucidum* a potential source of natural antimicrobial compounds as an alternative or supplement to current antimicrobial treatments, opening important therapeutic perspectives. Further studies are needed to isolate the bioactive compounds from *G. lucidum* extracts to elucidate the exact mechanism of action for their possible role in the treatment of infectious diseases. Further work is also required to fully understand how the extracts of *G. lucidum* interact with mammalian cells.

## Figures and Tables

**Table 1:** Position and description of selective sweeps in control *S. aureus*.

Position	Annotations	Genes	Products
1,886,098	K4 N * (AAA→AAC)	*KQ76_RS09235*	DUF1433 domain-containing protein
2,574,726	G69 A * (GGC→GCC)	*KQ76_RS13020*	alpha/beta hydrolase
2,389,188	E187 Q * (GAA→CAA)	*hssR*	DNA-binding heme response regulator
2,389,192	R188 P * (CGA→CCA)	*hssR*	DNA-binding heme response regulator
2,574,727	G69 R * (GGC→CGC)	*KQ76_RS13020*	alpha/beta hydrolase
2,190,680	T500 S * (ACG→TCG)	*KQ76_RS10985*	BglG family transcription antiterminator
2,776,116	H117 Q * (CAT→CAA)	*mnmG*	tRNA uridine-5-carboxymethylaminomethyl(34) synthesis enzyme
2,564,193	M16 I * (ATG→ATC)	*KQ76_RS12955*	D-lactate dehydrogenase
2,564,194	A17 P * (GCA→CCA)	*KQ76_RS12955*	D-lactate dehydrogenase
986,858	Q59 K * (CAA→AAA)	*KQ76_RS04770*	ATP-binding protein
1,925,247	A282 P * (GCC→CCC)	*KQ76_RS09465*	exonuclease SbcCD subunit D
2,574,728	E68 D * (GAG→GAC)	*KQ76_RS13020*	alpha/beta hydrolase
2,252,747	Intergenic (−54/+157)	*KQ76_RS11280/KQ76_RS11285*	M23 family metallopeptidase/HAD-IIB family hydrolase
2,389,185	D186 H * (GAT→CAT)	*hssR*	DNA-binding heme response regulator
2,665,478	E162 V * (GAA→GTA)	*KQ76_RS13475*	glutathione peroxidase
2,189,216	A12 P * (GCC→CCC)	*KQ76_RS10985*	BglG family transcription antiterminator
2,389,198	V190 G * (GTT→GGT)	*hssR*	DNA-binding heme response regulator
14,600	Intergenic (+504/−140)	*serS/KQ76_RS00050*	serine--tRNA ligase/AzlC family ABC transporter permease
1,890,405	S137 T * (AGT→ACT)	*KQ76_RS09255*	hypothetical protein
1,547,921	S147 T * (AGT→ACT)	*KQ76_RS07500*	conserved phage C-terminal domain-containing protein

* Annotation gives meaning to a given sequence and makes it much easier to view and analyze its contents.

**Table 2:** Position and description of selective sweeps in *S. aureus* cells treated with ethanol.

Position	Annotations	Genes	Products
1,886,098	K4 N * (AAA→AAC)	*KQ76_RS09235*	DUF1433 domain-containing protein
2,189,216	A12 P * (GCC→CCC)	*KQ76_RS10985*	BglG family transcription antiterminator
2,389,192	R188 P * (CGA→CCA)	*hssR*	DNA-binding heme response regulator
2,574,726	G69 A (GGC→GCC)	*KQ76_RS13020*	alpha/beta hydrolase
986,858	Q59 K * (CAA→AAA)	*KQ76_RS04770*	ATP-binding protein
2,746,925	A172 G * (GCT→GGT)	*KQ76_RS13825*	ECF-type riboflavin transporter substrate-binding protein
673,306	A185 P * (GCA→CCA)	*graR*	response regulator transcription factor GraR/ApsR
2,389,188	E187 Q * (GAA→CAA)	*hssR*	DNA-binding heme response regulator
2,574,727	G69 R * (GGC→CGC)	*KQ76_RS13020*	alpha/beta hydrolase
2,541,135	Y291 * (TAC→TAG)	*gntK*	gluconokinase
1,213,003	E184 D * (GAG→GAC)	*ylqF*	ribosome biogenesis GTPase
986,867	H62 N * (CAT→AAT)	*KQ76_RS04770*	ATP-binding protein
2,776,116	H117 Q * (CAT→CAA)	*mnmG*	tRNA uridine-5-carboxymethylaminomethyl(34) synthesis enzyme
453,752	pseudogene (4457/4500 nt)	*gltB*	glutamate synthase large subunit
2,389,185	D186 H * (GAT→CAT)	*hssR*	DNA-binding heme response regulator
1,890,405	S137 T * (AGT→ACT)	*KQ76_RS09255*	hypothetical protein
2,252,747	intergenic (−54/+157)	*KQ76_RS11280/KQ76_RS11285*	M23 family metallopeptidase/HAD-IIB family hydrolase
853,282	E34 Q * (GAA→CAA)	*lipA*	lipoyl synthase
986,873	E64 * (GAA→TAA)	*KQ76_RS04770*	ATP-binding protein
25,274	E93 Q * (GAA→CAA)	*mco*	multicopper oxidase

* Annotation gives meaning to a given sequence and makes it much easier to view and analyze its contents.

**Table 3:** Position and description of selective sweeps in *S. aureus* cells treated with aqueous extract of *G. lucidum*.

Position	Annotations	Genes	Products
1,886,098	K4 N * (AAA→AAC)	*KQ76_RS09235*	DUF1433 domain-containing protein
2,189,216	A12 P * (GCC→CCC)	*KQ76_RS10985*	BglG family transcription antiterminator
2,389,192	R188 P * (CGA→CCA)	*hssR*	DNA-binding heme response regulator
2,574,726	G69 A * (GGC→GCC)	*KQ76_RS13020*	alpha/beta hydrolase
986,858	Q59 K * (CAA→AAA)	*KQ76_RS04770*	ATP-binding protein
2,746,925	A172 G * (GCT→GGT)	*KQ76_RS13825*	ECF-type riboflavin transporter substrate-binding protein
673,306	A185 P * (GCA→CCA)	*graR*	response regulator transcription factor GraR/ApsR
2,389,188	E187 Q * (GAA→CAA)	*hssR*	DNA-binding heme response regulator
2,574,727	G69 R (GGC→CGC)	*KQ76_RS13020*	alpha/beta hydrolase
2,541,135	Y291 * (TAC→TAG)	*gntK*	gluconokinase
1,213,003	E184 D * (GAG→GAC)	*ylqF*	ribosome biogenesis GTPase
986,867	H62 N * (CAT→AAT)	*KQ76_RS04770*	ATP-binding protein
2,776,116	H117 Q * (CAT→CAA)	*mnmG*	tRNA uridine-5-carboxymethylaminomethyl(34) synthesis enzyme
453,752	pseudogene (4457/4500 nt)	*gltB*	glutamate synthase large subunit
2,389,185	D186 H * (GAT→CAT)	*hssR*	DNA-binding heme response regulator
1,890,405	S137 T * (AGT→ACT)	*KQ76_RS09255*	hypothetical protein
2,252,747	intergenic (−54/+157)	*KQ76_RS11280/KQ76_RS11285*	M23 family metallopeptidase/HAD-IIB family hydrolase
853,282	E34 Q * (GAA→CAA)	*lipA*	lipoyl synthase
986,873	E64 * (GAA→TAA)	*KQ76_RS04770*	ATP-binding protein
25,274	E93 Q * (GAA→CAA)	*mco*	multicopper oxidase

* Annotation gives meaning to a given sequence and makes it much easier to view and analyze its contents.

**Table 4:** Position and description of selective sweeps in *S. aureus* cells treated with dual solvent extractions of *G. lucidum*.

Position	Annotations	Genes	Products
1,886,098	K4 N * (AAA→AAC)	*KQ76_RS09235*	DUF1433 domain-containing protein
2,190,680	T500 S * (ACG→TCG)	*KQ76_RS10985*	BglG family transcription antiterminator
2,389,192	R188 P * (CGA→CCA)	*hssR*	DNA-binding heme response regulator
2,574,726	G69 A * (GGC→GCC)	*KQ76_RS13020*	alpha/beta hydrolase
2,746,925	A172 G * (GCT→GGT)	*KQ76_RS13825*	ECF-type riboflavin transporter substrate-binding protein
2,564,194	A17 P * (GCA→CCA)	*KQ76_RS12955*	D-lactate dehydrogenase
2,389,185	D186 H * (GAT→CAT)	*hssR*	DNA-binding heme response regulator
2,389,188	E187 Q * (GAA→CAA)	*hssR*	DNA-binding heme response regulator
1,827,481	A30 P * (GCA→CCA)	*sapep*	Mn(2+)-dependent dipeptidase
2,776,116	H117 Q * (CAT→CAA)	*mnmG*	tRNA uridine-5-carboxymethylaminomethyl(34) synthesis enzyme
1,213,003	E184 D * (GAG→GAC)	*ylqF*	ribosome biogenesis GTPase
2,189,216	A12 P * (GCC→CCC)	*KQ76_RS10985*	BglG family transcription antiterminator
2,665,478	E162 V * (GAA→GTA)	*KQ76_RS13475*	glutathione peroxidase
14,600	intergenic (+504/−140)	*serS/KQ76_RS00050*	serine--tRNA ligase/AzlC family ABC transporter permease
2,233,551	T39 S * (ACT→AGT)	*KQ76_RS11175*	BCCT family transporter
460,550	intergenic (+70/−69)	*KQ76_RS02145/KQ76_RS02150*	hypothetical protein/N-acetyltransferase
2,541,135	Y291 * (TAC→TAG)	*gntK*	gluconokinase
2,574,727	G69 R * (GGC→CGC)	*KQ76_RS13020*	alpha/beta hydrolase
986,867	H62 N * (CAT→AAT)	*KQ76_RS04770*	ATP-binding protein
2,291,741	intergenic (−5/+112)	*KQ76_RS11540/femX*	efflux RND transporter permease subunit/lipid II:glycine glycyltransferase

* Annotation gives meaning to a given sequence and makes it much easier to view and analyze its contents.

**Table 5:** Position and description of selective sweeps in *E. coli* control cells.

Position	Annotations	Genes	Products
5,022,977	L28 V (CTC→GTC)	*pqiB*	intermembrane transport protein
82,367	A100 P (GCC→CCC)	*mdoG*	glucans biosynthesis protein
5,022,975	A27 G (GCG→GGG)	*pqiB*	intermembrane transport protein
4,935,212	intergenic (−362/+132)	*lysO/aqpZ*	L-lysine exporter LysO/aquaporin
325,706	S9 R (AGC→AGG)	*sirB2*	invasion regulator SirB2
1,664,261	T163 T (ACC→ACG)	*fryC*	PTS fructose transporter subunit IIC
1,244,541	V392 L (GTA→CTA)	*hisD*	histidinol dehydrogenase
1,078,228	A53 P (GCC→CCC)	*D1792_RS05680*	DUF4756 family protein
4,935,163	intergenic (−313/+181)	*lysO/aqpZ*	L-lysine exporter LysO/aquaporin
2,077,731	intergenic (−7/+65)	*D1792_RS10070/D1792_RS10075*	phosphoglycerate dehydrogenase/SIS domain-containing protein
2,376,513	G116 G (GGC→GGA)	*D1792_RS11465*	YtfJ family protein
2,376,506	E119* (GAA→TAA)	*D1792_RS11465*	YtfJ family protein
2,725,980	G94 G (GGC→GGG)	*chiA*	bifunctional chitinase/lysozyme
2,936,459	L94* (TTA→TGA)	*rcdB*	LysR family transcriptional regulator
2,555,115	V98 L (GTG→CTG)	*diaA*	DnaA initiator-associating protein
4,935,182	intergenic (−332/+162)	*lysO/aqpZ*	L-lysine exporter LysO/aquaporin
2,936,458	L94 V (TTA→GTA)	*rcdB*	LysR family transcriptional regulator
2,077,733	intergenic (−9/+63)	*D1792_RS10070/D1792_RS10075*	phosphoglycerate dehydrogenase/SIS domain-containing protein

* Annotation gives meaning to a given sequence and makes it much easier to view and analyze its contents.

**Table 6:** Position and description of selective sweeps in *E. coli* cells treated with ethanol extract of *G. lucidum*.

Position	Annotations	Genes	Products
3,963,240	T278 P * (ACC→CCC)	*lgoR*	GntR family transcriptional regulator
2,376,513	G116 G * (GGC→GGA)	*D1792_RS11465*	YtfJ family protein
325,706	S9 R * (AGC→AGG)	*sirB2*	invasion regulator SirB2
5,022,977	L28 V * (CTC→GTC)	*pqiB*	intermembrane transport protein
82,367	A100 P * (GCC→CCC)	*mdoG*	glucans biosynthesis protein
1,500,935	E15 D * (GAA→GAC)	*ubiG*	bifunctional 2-polyprenyl-6-hydroxyphenol methylase/3-demethylubiquinol 3-O-methyltransferase UbiG
1,664,262	T163 S * (ACC→AGC)	*fryC*	PTS fructose transporter subunit IIC
1,997,599	G22 G * (GGG→GGC)	*ygcS*	MFS transporter
4,333,274	intergenic (−1072/−34)	*pic/D1792_RS20755*	serine protease autotransporter toxin Pic/hypothetical protein
4,333,275	intergenic (−1073/−33)	*pic/D1792_RS20755*	serine protease autotransporter toxin Pic/hypothetical protein
4,333,278	intergenic (−1076/−30)	*pic/D1792_RS20755*	serine protease autotransporter toxin Pic/hypothetical protein
4,333,296	intergenic (−1094/−12)	*pic/D1792_RS20755*	serine protease autotransporter toxin Pic/hypothetical protein
4,333,282	intergenic (−1080/−26)	*pic/D1792_RS20755*	serine protease autotransporter toxin Pic/hypothetical protein
4,333,283	intergenic (−1081/−25)	*pic/D1792_RS20755*	serine protease autotransporter toxin Pic/hypothetical protein

* Annotation gives meaning to a given sequence and makes it much easier to view and analyze its contents.

**Table 7:** Position and description of selective sweeps in *E. coli* cells treated with dual solvent extractions of *G. lucidum*.

Position	Annotations	Genes	Products
461,348	E158 D * (GAG→GAC)	*D1792_RS02575*	helix-turn-helix transcriptional regulator
2,376,513	G116 G * (GGC→GGA)	*D1792_RS11465*	YtfJ family protein
1,313,879	F3 Y * (TTC→TAC)	*baeS*	two-component system sensor histidine kinase
5,022,977	L28 V * (CTC→GTC)	*pqiB*	intermembrane transport protein
4,333,240	intergenic (−1038/−68)	*pic/D1792_RS20755*	serine protease autotransporter toxin Pic/hypothetical protein
1,244,541	V392 L * (GTA→CTA)	*hisD*	histidinol dehydrogenase

* Annotation gives meaning to a given sequence and makes it much easier to view and analyze its contents.

## Data Availability

Data are contained within the article.
